# Operative View of a Giant Pedunculated, Fistulizing Hydatid Cyst of the Liver

**DOI:** 10.4269/ajtmh.23-0598

**Published:** 2023-10-23

**Authors:** Anis Hasnaoui, Houda Kammoun, Racem Trigui

**Affiliations:** ^1^ Department of General Surgery, Menzel Bourguiba Hospital, Bizerta, Tunisia;; ^2^ Faculty of Medicine of Tunis, Tunis El Manar University, Tunis, Tunisia

**Figure 1.**
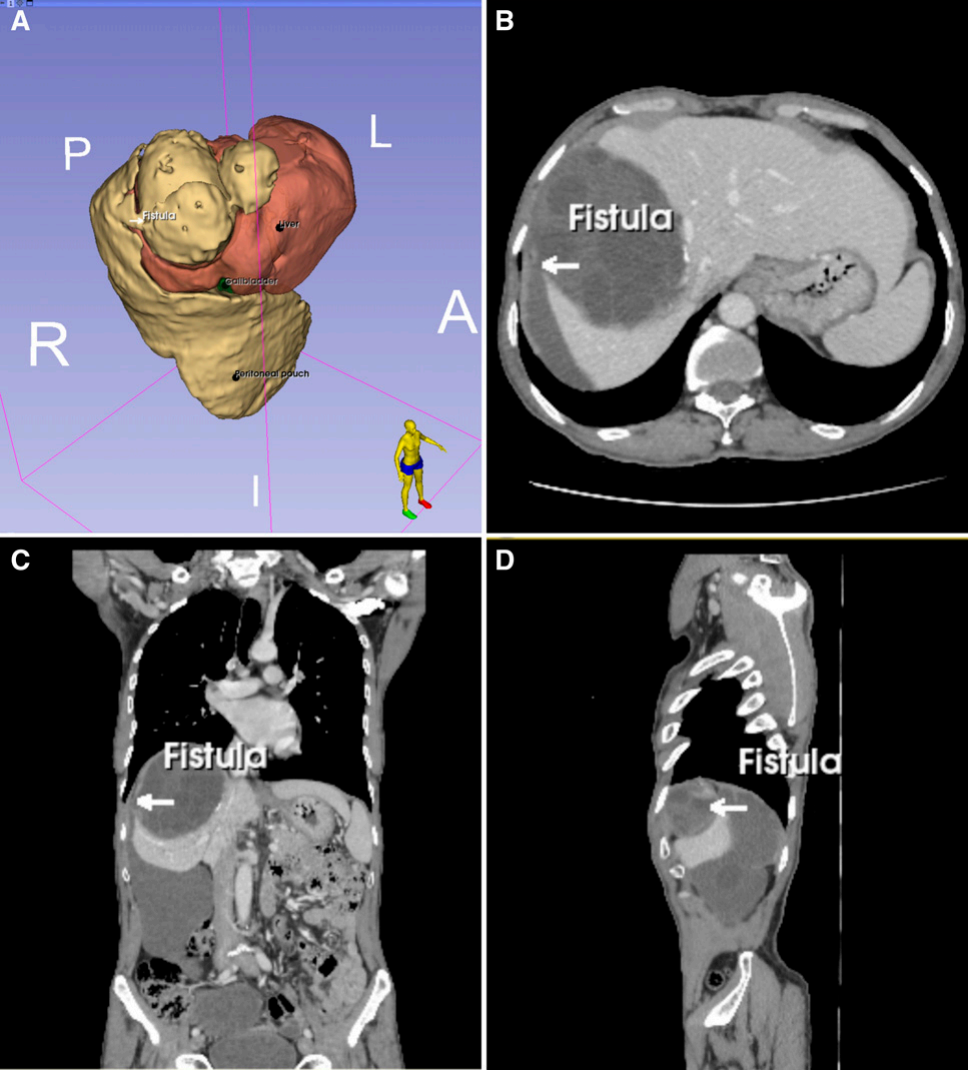
Computed tomography highlighting the fistulous path (white arrows). Three-dimensional reconstruction (**A**), axial view (**B**), coronal view (**C**), and sagittal view (**D**) showing the hepatic hydatid cyst and the pedunculated cyst in the right paracolic gutter, highlighting the fistulous path between them.

**Figure 2.**
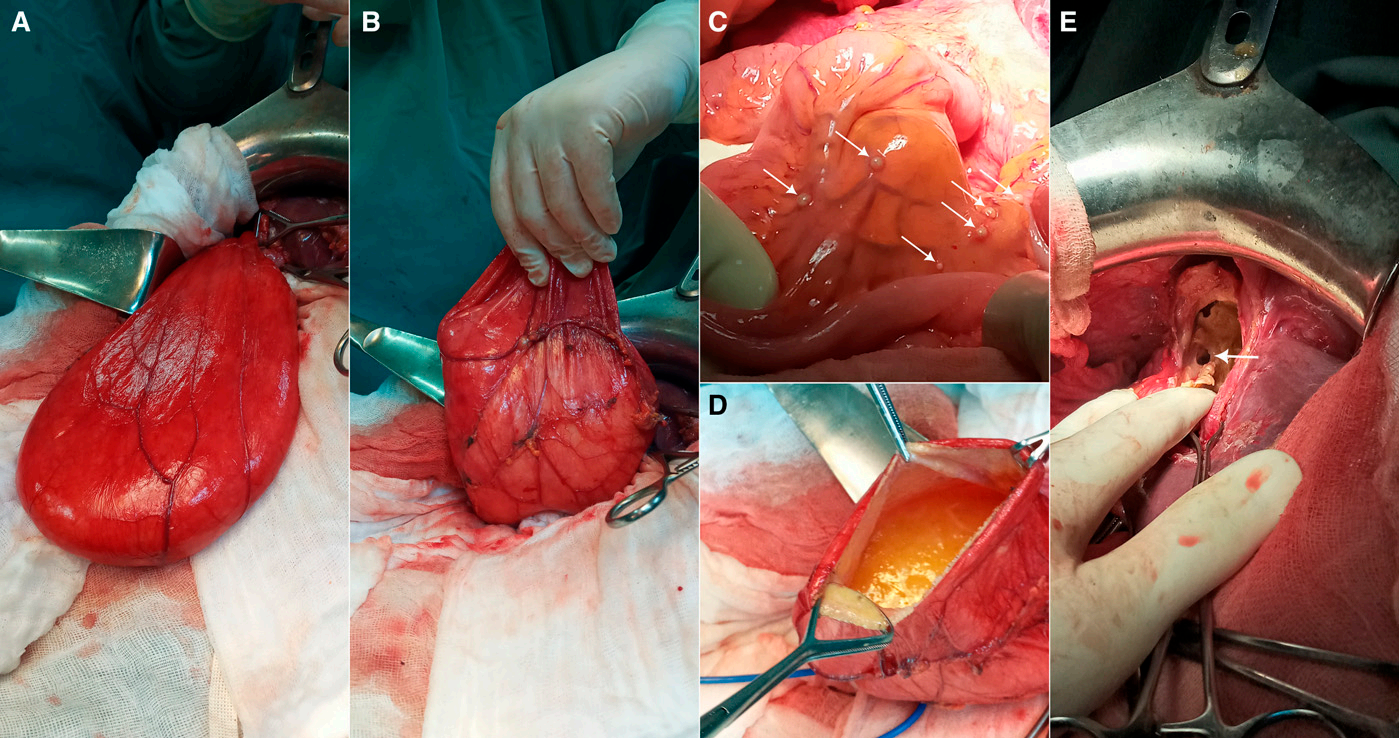
Intraoperative findings. (**A**, **B**) A giant pedunculated hydatid cyst dissected out of the abdomen and remaining attached only to the protruding dome of a hydatid cyst in the liver. (**C**) White arrows point to the peritoneal nodules. (**D**) The pedunculated cyst containing gelatinous fluid with several daughter cysts. (**E**) Intraoperative view of the fistulous path (white arrow) between the hepatic hydatid cyst and its pedunculated component.

A 65-year-old man presented with 5 months of persistent pain in the right hypochondrium without fever or jaundice. Physical examination and routine blood tests were normal. Abdominal ultrasound showed three hepatic cystic masses with morphologies suggestive of hydatid disease. Computed tomography unveiled an astonishing tableau of hepatic and peritoneal hydatidosis, with a voluminous fluid collection stretching over 27 cm within the right paracolic gutter. This expansive formation communicated with one of the hepatic cysts through a fistula (Figure 1). At laparotomy, a giant lesion was seen that traced a path from the right paracolic gutter to the protruding apex of a hydatid cyst nestled in the posterior section of the liver (Figure 2A and B). The pedunculated cyst contained gelatinous fluid with several daughter cysts (Figure 2D). Numerous, dispersed white nodules across the peritoneal cavity (Figure 2C) were seen, accompanied by a substantial 6-cm retro-vesical cyst.

After resection of the protruding domes of the hepatic cysts, the fistulous path between the hepatic cyst and the pedunculated peritoneal cyst was resected (Figure 2E). The postoperative course was uneventful, and the patient was initiated on albendazole in an attempt to prevent the formation of new hydatid cysts, in case spillage of cyst contents had occurred.

Hydatid disease, caused by the larval form of *Echinococcus granulosus*, is endemic in the Mediterranean area and elsewhere.^1^ This case highlights how a fistulous process relates to the natural history of hydatidosis called exo-vesiculation that could eventually cause peritoneal or retroperitoneal hydatidosis. Secondary peritoneal cysts are generally a result of spontaneous or traumatic rupture of liver cysts.^2^ When surgery is indicated, it is optimal to manage all cysts at once, if possible, to forestall potential complications.^3^ In the absence of clear recommendations, we opted for conservative surgery associated with postsurgical drug treatment with albendazole.^4^
